# Age, gender, personality, ideological attitudes and individual differences in a person's news spectrum: how many and who might be prone to “filter bubbles” and “echo chambers” online?

**DOI:** 10.1016/j.heliyon.2020.e03214

**Published:** 2020-01-17

**Authors:** Cornelia Sindermann, Jon D. Elhai, Morten Moshagen, Christian Montag

**Affiliations:** a Molecular Psychology, Institute of Psychology and Education, Ulm University, Ulm, 89081, Germany; b Department of Psychology, Department of Psychiatry, University of Toledo, Toledo, OH, USA; c Research Methods, Institute of Psychology and Education, Ulm University, Ulm, 89081, Germany

**Keywords:** Psychology, Media psychology, Individual differences, Digital media, Political science, Political behavior, News spectrum, Big Five, RWA, Filter bubble, Echo chamber

## Abstract

Potential effects of demographics, personality, and ideological attitudes on the number of news sources consumed should be investigated. The number of news sources consumed, in turn, was seen as inverse proxy for the susceptibility to be caught in “filter bubbles” and/or “echo chambers” (online), which are hotly discussed topics also in politics. A sample of 1,681 (*n* = 557 males) participants provided data on demographics, the Big Five as well as Right-Wing Authoritarianism (RWA) alongside the number of different news sources consumed and current voting preferences. Results showed that age (positively), gender (higher in males), Openness (positively), and RWA (negatively) predicted the number of different news sources consumed. The group of participants consuming news exclusively offline showed highest scores in Conscientiousness and lowest scores in Neuroticism compared to the “news feeds only” and the “news feeds and online” groups. However, less than 5% of the participants exclusively consumed news via news feeds of social networking sites. Participants who stated that they would not vote reported the lowest number of different news sources consumed. These findings reveal first insights into predisposing factors for the susceptibility to be caught in “filter bubbles” and/or “echo chamber” online and how this might be associated with voting preferences.

## 1 Introduction

Within the past years the personalization of Internet applications, such as receiving personalized news content, has represented an important digital progress. Despite several possible advantages, also criticism has emerged. In this realm, terms such as “filter bubble” and “echo chamber” in relation to online news consumption have gained special attention in the popular press as well as in science. In the present work, the role of demographic variables, individual differences in personality, and ideological attitudes will be investigated in light of news consumption and therefore also in light of potential risks to being caught in “filter bubbles” and “echo chambers”.

The term “filter bubble” refers to a potential and extreme consequence of pre-selected/implicit personalization (not driven by the user itself) of the Internet (Thurman and Schifferes, 2012; Zuiderveen Borgesius et al., 2016) and was first mentioned by Pariser (2011). It describes the consequences of prediction engines (i.e. algorithms) on the Internet constantly analyzing various data points of the individual and creating different sets of information presented to each individual, accordingly (Pariser, 2011). Such algorithms are used in a wide range of Internet sites; for example, the search engine Google and the social networking site (including its news feed) Facebook, to name but a few (https://www.google.com/intl/en_uk/search/howsearchworks/; https://newsroom.fb.com/news/2019/03/why-am-i-seeing-this/). The personalized, selective, and varying information such algorithms present to each person separately can lead to the so called “filter bubble”, that individuals fall for (often without knowing) (Pariser, 2011). The term “filter bubble” should draw attention to the following problems of the pre-selected personalization: i) people are alone in their personalized information bubble, ii) the bubble is invisible; hence, people mostly do not know if and/or what kind of information about themselves is collected and analyzed; this potentially leads to the misbelief that the presented set of information is unbiased, iii) people do not choose to enter the “filter bubble” actively but are put into it passively (Pariser, 2011).

The term “echo chamber” is a metaphor for an environment in which a person is exposed only to certain information again and again (Jamieson and Cappella, 2008). Such an environment is thought to lead to a reinforcement of one's attitudes when attitude-fitting information (e.g. news, opinions, beliefs) is repeated and amplificated while counter-attitudinal information is missing (Jamieson and Cappella, 2008). This, ultimately can lead to group polarization (Stroud, 2010; Sunstein, 2002). One easy way to create an “echo chamber” seems to be joining a social group of individuals, who have common beliefs and opinions, on online social media platforms (such as Facebook). Accordingly, the term “echo chamber” is often discussed as an extreme consequence of self-selected/explicit personalization (driven by the users itself) of the Internet (Barberá et al., 2015; Zuiderveen Borgesius et al., 2016). One might argue that creation of an “echo chamber” is also possible in the offline world by only consuming certain TV channels or newspapers. However, we argue that creation of an “echo chamber” on the Internet is easy. For example, the Internet is a high choice environment and provides the possibility of meeting many individuals from all over the world, breaking local restrictions. In conclusion, users of social media platforms and social networking sites seem to be at risk for both “filter bubbles” as well as “echo chambers”.

Supporting evidence for the existence of selective information presentation and consumption, hence “filter bubbles” and “echo chambers”, on the Internet comes from several studies. As an example, one study reports that systems preselecting/implicitly selecting personalized information can indeed lead to diminished presentation and consumption of counter-attitudinal information (Beam, 2014). Moreover, one study observed that around 12% of Google's web search results show differences between users, which can be explained by pre-selected/implicit personalization (Hannak et al., 2013). Both studies support the “filter bubble” hypothesis. Another study (among others) demonstrated that individuals indeed choose to read news items that seemingly are in line with their own opinion (Garrett, 2009). However, the effect regarding avoidance of counter-attitudinal news items was reported to be less striking in this study (Garrett, 2009). Additionally, studies by Iyengar and Hahn (2009) and by Peterson et al. (2018) indicate that individuals prefer to read news stories, news websites, and content fitting with their own political orientation. These findings further underline the “echo chamber” hypothesis.

The potential of the limited and skewed presentation and consumption of online information is critically discussed worldwide. Specifically, some researchers fear that the personalization of diverse Internet platforms resulting in “filter bubbles” and/or “echo chambers” could have tremendous consequences, in particular when information about news and political campaigns are at stake. As such, it is also feared that “filter bubbles” and “echo chambers” have the potential to undermine democracy (Bozdag & van den Hoven, 2015; Sunstein, 2004). In accordance with this fear, a study by Epstein and Robertson (2015) found that biased rankings in web-search results can influence voting preferences of undecided voters.

Despite the criticism and fears of personalization of the Internet, it seems clear that the abundant knowledge available online makes it necessary to rely on algorithms filtering information (the algorithms themselves clearly need to be discussed). Moreover, the tendency to seek information consistent with one's attitudes is not necessarily negative. Additionally, the actual extent of “filter bubbles” and “echo chambers” is questionable. In line with this, a study by Flaxman et al. (2016) on web-browsing records, reports that articles found via social media as well as search engines indeed showed higher ideological segregation scores than articles found by direct browsing on a news page online. However, social media and search engines were also linked to a higher exposure to politically diverse, hence, also opposing information. This result indicates that individuals are still confronted with counter-attitudinal information on social media platforms and search engines online. In line with this finding, several more studies indicate that, while indeed being exposed to more attitudinal-fitting information, individuals are also exposed to counter-attitudinal information on social media platforms, which are highly discussed in light of “filter bubbles” as well as “echo chambers” (Bakshy et al., 2015; Newman et al., 2017). Additionally, one study investigating 14 days of news feed content of 1.000 Danes estimates that on Facebook only 10–27.8% are caught in a “filter bubble” (Bechmann and Nielbo, 2018).

In addition to the discussion on the extent and consequences of personalization of the Internet, another important topic is rarely considered in most of the studies about “filter bubbles” and “echo chambers”: The individual person him/herself. Independent of the effect of potential online “filter bubbles” and “echo chambers” on information consumption, the person is the one to decide whether or not to completely expose him-/herself to information solely presented online; or even more extreme, on social networking sites, only. As an example and important thought for the present study: We argue that individuals who only consume news via news feeds or social groups on social media platforms might be more prone to end up in a “filter bubble” and/or an “echo chamber” compared to individuals who (also) consume news offline such as via TV news, printed newspapers or radio. Expanding this idea, a recent study found that media diversity was negatively associated with being caught in an “echo chamber” (Dubois and Blank, 2018). An important factor, which might contribute to the decision about which and how many different kinds of news sources are consumed, is personality.

According to the classic Five-Factor Model of Personality, personality is organized into five broad domains. Today these factors are commonly referred to as Extraversion (socially active and assertive), Agreeableness (altruistic, compliant), Conscientiousness (orderly, self-disciplined), Neuroticism (anxious, depressed/worried), and Openness (to experience) (being open for and interested in new ideas, aesthetics). Together these factors are commonly known as the Big Five (Digman, 1990; Rammstedt and Danner, 2017). These terms are also used in the self-report measure applied in the present study, which is why the descriptions of the Big Five factors mentioned above are tailored to this self-report measure (Rammstedt and Danner, 2017). Previous literature indicates that Extraversion is higher and Conscientiousness is lower in users of social networking sites/messenger applications than in non-users (Brailovskaia and Margraf, 2016; Eşkisu et al., 2017; Montag et al., 2015; Ryan and Xenos, 2011; Wehrli, 2008). Moreover, studies indicate that females might use social networking sites more than males do (Brailovskaia and Margraf, 2016; Ryan and Xenos, 2011). Lastly, age was negatively associated with usage of social networking sites/messenger applications in several studies (but not all studies tested for statistical significance) (Brailovskaia and Margraf, 2016; Montag et al., 2015; Ryan and Xenos, 2011; Wehrli, 2008). These findings lead to the conclusion that Extraversion, Conscientiousness, gender, and age might also be associated with the probability of reading news (exclusively) on social networking sites and therefore to be at higher or lower risk to end up in a “filter bubble” and/or an “echo chamber”.

Another, potentially important, factor in relation to information consumption is the ideological attitude of Right-Wing Authoritarianism (RWA). This ideological attitude is described as the extent to which individuals tend to i) adhere to conventional values, ii) be submissive to in-group authorities, and iii) show aggression towards individuals who violate conventional values or who are punished by in-group authorities (Adorno et al., 1950; Aichholzer and Zeglovits, 2015; Altemeyer, 1996). A study on intolerance reports that RWA was among other factors found to positively predict political intolerance with respect to various activists for, for example, gay and abortion rights as well as affirmative action. Also intolerance towards an immigrant rights group was found to be positively predicted by RWA, if this group was expected to gain power and status (Crawford and Pilanski, 2014). Moreover, another study found that RWA was positively related to the perceived veracity and negatively related to perceived author bias of news reports arguing against same-sex relationships (whereas it was negatively related to perceived veracity of an article arguing pro same-sex relationships) (Crawford et al., 2013). This finding is notable as other studies report that RWA is generally associated with a negative attitude and prejudice toward homosexual individuals (Crawford et al., 2016; Rowatt et al., 2009). Additionally, RWA seems to be associated with selective interest in information fitting to one's own attitudes in a threat condition, which in turn might lead to stronger opinions and more resistance to attitudinal changes (Lavine et al., 2005). Given these empirical results and the defining characteristic of negativity towards individuals who do not share the same opinions (Adorno et al., 1950; Aichholzer and Zandonella, 2016), it seems plausible to assume that RWA might be especially associated with selective news consumption (fitting to one's own pre-existing attitudes, opinions, and beliefs).

Moreover, age was among other variables found to be positively associated with interest in keeping up with recent news and news exposure via TV (Chyi and Lee, 2013; Wonneberger et al., 2012). Given these findings as well as findings on associations between age and usage of social networking sites/messenger applications mentioned above, also potential effects of age on the number of different news sources consumed were investigated in the present work. Lastly, a study reports (and further investigates) that females show less news consumption compared to males (Benesch, 2012). Given these findings and results on gender associations with social networking site use mentioned above, it was decided to also include gender in the analyses of the present work.

In conclusion, the present study aims at investigating whether and to what extent demographic variables, personality, and one's own ideological attitude might contribute to the decision about which and how many different news sources are used to obtain information about news. The basic idea is: The more different news sources an individual consumes, the lower the risk to end up in a “filter bubble” and/or “echo chamber”. On the other hand, consuming news exclusively via online social networking sites is thought be associated with the highest risk to end up in a “filter bubble” and/or “echo chamber”. Additional analyses on associations with current voting preferences in Germany are also presented. Given the lack of available literature on the present topic, it should also be mentioned that the present study has an exploratory characteristic.

## 2 Materials and methods

### 2.1 Participants

The data were collected anonymously via an online-platform (SurveyCoder by Christopher Kannen: https://ckannen.com/) on media usage between August 2018 and June 2019. Of note, European elections were in May 2019 and therefore fell within the timeframe of data collection. The platform was advertised by several media outlets (TV, radio, press, and Internet) to be able to recruit a heterogeneous sample. In more detail: When a researcher of our group gave an interview (in a newspaper, TV show, …), the link to the survey was also shown and the rationale was explained so interested individuals could participate. All participants were provided with information on their personality and smartphone use as an incentive to join the present research project. Other self-report measures (not mentioned here) were also assessed, but are not relevant to the present project (detailed information uploaded at the OSF: https://osf.io/jxqvs/files/). The study was approved by the local ethics committee of Ulm University, Ulm, Germany. All participants provided informed electronic consent prior to participation. Underaged participants were asked to obtain consent from their parents/legal guardians.

A total of *N* = 1,894 German-speaking participants took part in the present online study. After data cleaning (please see Supplementary Material), a final sample of *N* = 1,681 participants (*n* = 557 males, *n* = 1,124 females) remained. The mean age of the sample was 34.44 years (*SD* = 15.09) with a range from 12 to 81 years and a median of 33 years. The sample partly overlaps with samples from other works on personality and Facebook use (*n* = 41), as well as Maslow's hierarchy of needs, personality, and primary emotional traits (*n* = 785) currently submitted to other scientific journals.

### 2.2 Materials

#### 2.2.1 Big five inventory

To assess individual differences on the Big Five personality variables, the German version of the Big Five Inventory (BFI) was used. The German version of the BFI includes one additional item in the scale assessing Agreeableness. Hence, in total 45 instead of 44 items were administered (Rammstedt and Danner, 2017). For better comparability with other studies, the additional item was not included in the present analyses. All items in this questionnaire are answered on a 5-point Likert-scale ranging from 1 = “very inapplicable” to 5 = “very applicable”. In addition to the five broad factors, two subscales for each factor can be calculated. However, this work will focus on the broad factors. Internal consistencies (using Cronbach's alpha) in the final sample of *N* = 1,681 participants were .86, .70, .83, .86, .78 for Extraversion, Agreeableness, Conscientiousness, Neuroticism, and Openness, respectively.

#### 2.2.2 Balanced short scale on authoritarian attitudes

To assess RWA, the balanced short scale on authoritarian attitudes was used (German: balancierte Kurzskala autoritärer Einstellungen; short: B-RWA-6) (Aichholzer and Zeglovits, 2015). It comprises 6 items answered on a 5-point Likert-scale from 1 = “applies very much” to 5 = “does not apply at all”. For calculating the mean score, the answer options were recoded: Higher values on the B-RWA-6 scale indicate higher authoritarian attitudes. Originally, one total score and three subscales can be calculated (each comprising two items). However, in this work it will be focused on the total score. Additionally, two answer options “I don't know” and “I refuse to answer” were presented to the participants. If a participant chose one of the latter two answer options for at least one item, the mean score was not calculated. Therefore, the mean score was available for *n* = 1,397 (*n* = 476 males) participants only. Internal consistency of the total scale was Cronbach's alpha = .66 in the present sample of *n* = 1,397 participants.

#### 2.2.3 Number of different news sources consumed

To assess the number of different news sources participants used to obtain information about recent news, they were first asked whether they watch/read/hear news on TV, in print media, on the radio, on online news websites, on their Facebook news feed, and on news feeds of other social networking sites (answer options: no versus yes). If they stated doing so, they were further asked how many different news sources they consumed within the last six months prior to participation in the present study in each category. For the news feeds, participants who endorsed reading news feeds were further asked how often they look at the respective news feeds (Facebook, others) on a 4-point Likert-scale: “every day”, “every week”, “every month”, “more rarely”.

From the variables about whether and how many news sources participants consumed, aggregate scores were built. First, the numbers of all news sources consumed within the past six months prior to participation across all media channels were added; hence, via TV, print media, radio, online news websites, Facebook's news feed, news feeds of other social networking sites (= total number of news sources). Therefore, the following formula was used, wherein the variables “TV_Yes”, “Print_Yes”, “Radio_Yes”, “Online_News_Yes”, “Facebook_Yes”, and “Other_SNSs_Yes” (SNSs = Social Networking Sites) were coded 1 if participants stated to have consumed news via the respective channel, and 0 if participants did not:

total number (of news sources) = (TV_Yes × Count_TV) + (Print_Yes × Count_Print) + (Radio_Yes × Count_Radio) + (Online_News_Yes × Count_Online_News) + Facebook_Yes + Other_SNSs_Yes.

Moreover, summed scores of the number of news sources consumed offline (TV, print, radio) versus online (online news websites, Facebook's news feed, news feeds of other social networking sites) were calculated, accordingly. Results regarding these scores are presented in the Supplementary Material.

### 2.3 Statistical analyses

SPSS statistics version 25 was used for data cleaning and R Version 3.5.2 (R Core Team, 2018) and several R-packages (such as car (Fox and Weisberg, 2019), nnet (Venables and Ripley, 2002), psych (Revelle, 2018), pscl (Zeileis et al., 2008), reshape (Wickham, 2007)) for the statistical analyses.

All of the BFI and B-RWA-6 scales showed skewness and kurtosis of lower than +/- 1. Only the distribution of scores on the numbers of news sources consumed online showed higher skewness in the total sample (skewness = 1.07) and higher skewness and kurtosis in the female sample (skewness = 1.18, kurtosis = 1.26). Therefore, and in line with the rule of thumb by Miles and Shevlin (2001), normality could not be assumed for this variable. However, overall the deviations were rare and minor and the sample size was large.

Therefore, associations between the variables of interest (mentioned above) and age and gender were analyzed by means of Pearson correlations and *t*-tests (Welch's *t*-tests when necessary) (effects did not differ meaningfully when investigating them via parametric or the respective non-parametric tests).

In addition, a generalized linear model was conducted to predict the number of news sources consumed in total by age, gender, the BFI scales, and the B-RWA-6 scale. In more detail, a zero-inflated negative binomial model (using a log-link function) was implemented for the prediction of the news sources consumed in total. This model was chosen given the nature (count variable), the excessive zeros, and over-dispersion in the dependent variable (Elhai et al., 2008; Long, 1997; Rodrıguez, 2013). The respective analyses and results regarding the numbers of news sources consumed offline and online are presented in the Supplementary Material. In the Supplementary Material correlations between the BFI, the B-RWA-6 score, and the summed scores on news sources consumed in total, offline, and online are also presented.

**Figure 1:**
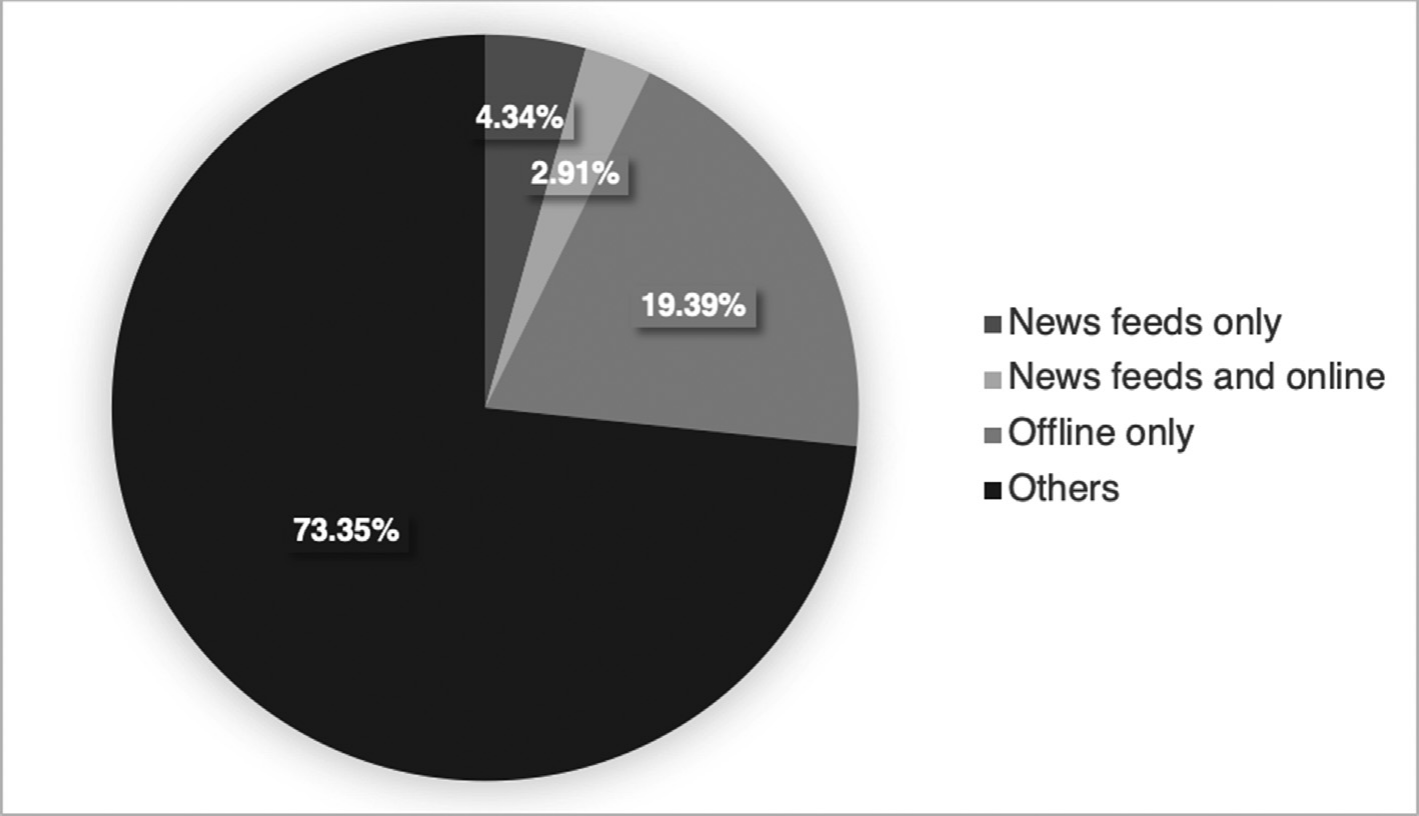
Distributions of groups consuming news via different channels in the total sample of *N* = 1,681 participants (percentages do not add up to exactly 100% due to rounding inaccuracies).

Lastly, three groups were formed. The first group comprised participants who only used news feeds of social networking sites (Facebook and/or others) to become informed about recent news within the past six months prior to participation (“news feeds only” group, *n* = 73/*n* *=* 49 with valid B-RWA-6 score). The next group comprised participants who used both news feeds of social networking sites and online news websites (“news feeds and online” group; *n* *=* 49/*n* = 46 with valid B-RWA-6 score). The last group comprised participants who exclusively used offline news sources to become informed about recent news (TV, print, radio) (“offline only”; *n* = 326/*n* = 272 with valid B-RWA-6 score). The reason to form a “news feeds only” group was that social media gains more and more attention as a news source (especially in younger individuals) (Newman et al., 2017) but in the same time the existence of both “filter bubbles” and “echo chambers” is in particular possible on these platforms. In line with this, it has been shown that personalized presentation of information is pronounced on social media, potentially because both pre- and self-selected personalization interact and potentiate their effects (Bakshy et al., 2015; Nikolov et al., 2015). However, both phenomena do not occur exclusively on such platforms. Accordingly, participants who also read news on online news websites are still at risk for both “filter bubbles” and “echo chambers”. But such participants also reduce their risk (as compared to the “news feeds only” group) by reading more diverse news sources online, which might, however, still be subject to preselected/implicit and self-selected/explicit personalization (however, potentially to a lower degree than social networking sites). Lastly, participants only consuming recent news offline might still be at risk of creating an “echo chamber” by self-selected/explicit personalization. However, their risk is lower and being put into a “filter bubble” is impossible. The distribution of participants in the different groups within the total sample is presented in Figure 1 (the groups of participants who used at least one online plus at least one offline source (*n* = 1050/*n* = 899 with valid B-RWA-6 score), who used only online news websites (*n* = 70/*n* = 58 with valid B-RWA-6 score), and who used none of the news sources (*n* = 113/*n* = 73 with valid B-RWA-6 score) to become informed about recent news are subsumed under the category “others”). The three groups (“news feeds only”, “news feeds and online”, “offline only”) were compared regarding their mean age with an ANOVA, and regarding their gender distribution by a *X*^2^-test. Additionally, a multinomial logistic regression was calculated to predict group membership (“news feeds only”, “news feeds and online”, “offline only”) by age, gender, the BFI, and B-RWA-6. The “news feeds only” group was used as reference group. To further investigate potential differences between each pair of these three groups on age, the BFI, and B-RWA-6 scales, a multivariate ANOVA and Tukey-Kramer post-hoc tests were calculated.

All presented *p*-values are derived from two-sided testing.

### 2.4 Additional analyses on current voting preferences

As can be seen from the present results, RWA was found to be an important negative predictor for the number of news sources consumed in total. Interesting findings on RWA indicate that it is associated with political orientation/ideology. Specifically, RWA is strongly interrelated with political conservatism (Duckitt et al., 2010; Jost et al., 2003). In line with this, RWA was found to be positively associated with preferences for the Republican party (versus the Democrats) in the US (Jost et al., 2009; Kemmelmeier, 2010; Smith and Winter, 2002). In Germany, however, the political system is more complex and more than only two parties are important. Therefore, it is of high interest to also investigate potential associations between the current voting preferences for German parties and both RWA (see Supplementary Material) and news consumption. Therefore, participants were asked which party they would vote for if general elections would be on the next Sunday. Answer options were “CDU/CSU” (English full name: Christian Democratic Union of Germany/Christian Social Union in Bavaria e.V.; European party: European People's Party; center(-right) parties), “SPD” (Social Democratic Party Germany; European party: Party of European Socialists; center-left party), “Grüne” (Green Party (complete name actually: Coalition 90/Green Party); European party: European Green Party; focused on environmental and social issues), “FDP” (Free Democratic Party; European party: Alliance of Liberals and Democrats for Europe; liberal party), “Linke” (Left Party; European party: Party of the European Left; left-wing-oriented party), “AfD” (Alternative for Germany; European party: European Alliance of Peoples and Nations; right-wing party), “others”, and “I would not vote” (https://www.britannica.com/place/Germany/Political-parties; https://www.expatica.com/de/about/gov-law-admin/the-main-political-parties-in-germany-107953/; https://www.dw.com/en/germanys-major-political-parties-what-you-need-to-know/g-43820148) (a more elaborate description is found in the Supplementary Material). In total *n* = 1,340 (*n* = 453 males, *n* = 887 females) participants, for which also the B-RWA-6 score was available, answered this voting preference question (of note: the item was only presented if the participants previously indicated that they were of German origin (versus from Switzerland, Austria or Liechtenstein)). The statistics on associations between the B-RWA-6 scores and voter groups are presented in the Supplementary Material alongside results on differences between voter groups in the Big Five. Additionally, a zero-inflated negative binomial model was implemented to investigate the effects of voter group, gender, and age on the number of news sources consumed in total (see significant associations of age and gender with the number of news sources consumed in total). Voter groups were dummy coded; the group of participants stating that they would not vote was used as reference group based on the descriptive statistics (lowest summed score of the number of news sources consumed in total).

## 3 Results

### 3.1 Associations with age and gender

Age correlated significantly with Extraversion (*r* = .06, *p* = .024), Agreeableness (*r* = .09, *p* < .001), Conscientiousness (*r* *=* *.*22, *p < .*001), Neuroticism (*r* = -.16, *p* < .001), Openness (*r* *=* *.*09, *p* < .001), and the summed score of consumed news sources in total (*r* *=* *.*28, *p < .*001).

Significant differences between males and females were found in Extraversion (*t*(1200.23) = -3.54, *p* < .001, Hedge's g = -.18), Agreeableness (*t*(1679) = -3.90, *p* < .001, Hedge's g = -.20), Conscientiousness (*t*(1679) = -4.72, *p* < .001; Hedge's g = -.24), and Neuroticism (*t*(1679) = -10.10, *p* < .001, Hedge's g = -.52). In all of these variables, males scored lower than females. Moreover, males scored significantly higher in the summed score of consumed news sources in total (*t*(971.16) = 5.74, *p* < .001, Hedge's g = .31). Descriptive statistics are presented in Table 1.

**Table 1:** Descriptive statistics in the total sample and split by gender.

	Total Sample (*N* = 1,681)^1^				Males (*n* = 557)^1^				Females (*n* = 1,124)^1^			
	Min	Max	M	SD	Min	Max	M	SD	Min	Max	M	SD
Extraversion	1.00	5.00	3.40	0.79	1.00	4.88	3.31	0.74	1.13	5.00	3.45	0.80
Agreeableness	1.78	5.00	3.53	0.56	2.00	5.00	3.45	0.54	1.78	5.00	3.57	0.56
Conscientiousness	1.22	5.00	3.57	0.68	1.33	5.00	3.46	0.67	1.22	5.00	3.62	0.68
Neuroticism	1.00	5.00	2.97	0.79	1.00	5.00	2.70	0.75	1.00	5.00	3.11	0.78
Openness	1.50	5.00	3.57	0.60	1.90	4.90	3.57	0.59	1.50	5.00	3.57	0.60
B-RWA-6^1^	1.00	4.67	2.73	0.67	1.00	4.50	2.71	0.67	1.00	4.67	2.74	0.67
Total number	0	24	7.88	5.24	0	24	8.97	5.72	0	23	7.35	4.90

*Note.* The row regarding total number refers to the summed score of numbers of news sources consumed in total. ^1^ Values of the B-RWA-6 (balanced short scale on authoritarian attitudes) are derived from the following sample sizes: *n*(total sample) = 1,397, *n*(males) = 476, *n*(females) = 921.

After Bonferroni-Holm adjustment (initial alpha level of 0.05) for multiple comparisons, of the significant associations between age and the variables of interest mentioned above, the correlation with Extraversion would not remain significant. After Bonferroni-Holm adjustment (initial alpha level of 0.05) on the gender differences, all significant differences reported above would still remain significant.

### 3.2 Investigating the news sources consumed in association with age, gender, personality, and ideological attitudes

#### 3.2.1 Predicting the number of news sources consumed in total

The zero-inflation model predicting excessive zeros shows that the intercept, age (Estimate = -1.03, *SE* = .233, *z* = -4.40, *p* < .001), and Conscientiousness (Estimate = -0.37, *SE* = .160, *z* = -2.30, *p* = .021) are significant predictors. This indicates that younger age and lower Conscientiousness are associated with an increased chance that zeros occur because individuals do not consume news at all. The (zero-inflated) negative binomial (count) model is presented in Table 2. As can be seen in this table, age (positively), gender (negatively: implicating higher scores for males), Openness (positively), and the B-RWA-6 scale (negatively) are significant predictors for the number of news sources consumed in total. The regression weights indicate that the predicted number of news sources consumed in total increases by 13% (exp(0.12) = 1.13) if age increases by one standard deviation (while holding all other variables constant), decreases by 19% (exp(-0.21) = 0.81) for being female (while holding all other variables constant), increases by 4% (exp(0.03) = 1.04) if Openness is increased by one standard deviation (while holding all other variables constant), and decreases by 5% (exp(-0.05) = 0.95) if the B-RWA-6 score increases by one standard deviation (while holding all other variables constant).

**Table 2:** (Zero-inflated) negative binomial model predicting the number of news sources consumed in total by age, gender, the Big Five, and RWA.

	Estimate	SE	z	p
Intercept	2.25	.028	79.60	<.001
Age	0.12	.017	7.05	<.001
Gender	-0.21	.036	-5.74	<.001
Extraversion	0.02	.018	0.95	.340
Agreeableness	-0.01	.018	-0.81	.418
Conscientiousness	-0.01	.018	-0.31	.759
Neuroticism	(-)0.00	.019	-0.09	.925
Openness	0.03	.017	2.03	.042
B-RWA-6	-0.05	.016	-2.79	.005

*Note. n* = 1,397. Gender was dummy coded as 0 = male, and 1 = female. The predictors (except gender) were z-standardized (in the complete sample) before including them in the model; hence, in *N* = 1,681 for age and the Big Five and in *n* = 1,397 for the B-RWA-6 (balanced short scale on authoritarian attitudes). Log(theta) = 1.57, *p* < .001.

#### 3.2.2 Investigating group membership

The mean age of the “news feeds only” group (*n* = 73) was *M* = 24.26 years (*SD* = 11.25). The mean age of the “news feeds and online” group (*n* = 49) was *M* = 26.57 years (*SD* = 10.42). And the mean age of the “offline only” group (*n* = 326) was *M* = 36.39 (*SD* = 16.18). An ANOVA revealed that the groups were significantly different on age (*F*(2,445) = 25.28, *p* < .001, η^2^(partial) = .102) (further results on pairwise comparisons of the groups, including participants for which the B-RWA-6 score was available, can be found below). The groups did not differ significantly in their gender distribution (*X*^2^ = 1.12, *p* = .570) (“news feeds only” (*n* = 73): 25% male, 75% female; “news feeds and online” (*n* = 49): 31% male, 69% female, “offline only” (*n* = 326): 24% male, 76% female).

**Figure 2:**
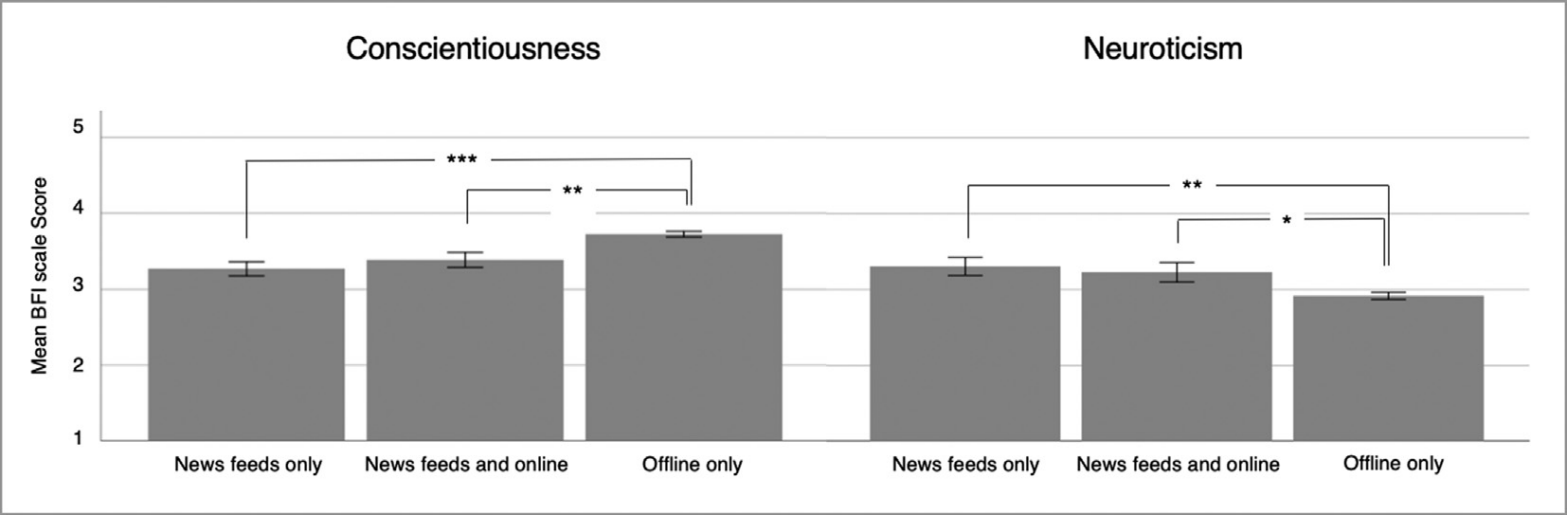
Mean Values and SEs (+/- 1 *SE*) in the “news feeds only” (*n* = 49), the “news feeds and online” (*n* = 46), and the “offline only” (*n* = 272) groups (only participants included, for which also the B-RWA-6 score was available); Descriptive statistics: Conscientiousness: *M* = 3.27 (*SD* = 0.65); *M* = 3.39 (*SD* = 0.68); *M* = 3.72 (*SD* = 0.63); Neuroticism: *M* = 3.30 (*SD* = 0.84); *M* = 3.23 (*SD* = 0.87); *M* = 2.91 (*SD* = 0.81); *p < .05, **p < .01, ***p < .001.

The multinomial logistic regression model (Table 3) showed that higher age, higher Conscientiousness, and lower Neuroticism significantly predicted being a member of the “offline only” group compared to the “news feeds only” group. However, no significant predictors were found for being a member of the “news feeds and online” group versus the “news feeds only” group. An additional multivariate ANOVA (dependent variables: age, BFI, and B-RWA-6 scales) also indicated significant differences between the three groups (Multivariate effect: *F*(14,718) = 5.24, *p* < .001). The groups differed significantly in age (*F*(2,364) = 23.58, *p* < .001 η^2^(partial) = .115), Conscientiousness (*F*(2,364) = 14.01, *p* < .001, η^2^(partial) = .071), and Neuroticism (*F*(2,364) = 6.60, *p* = .002, η^2^(partial) = .035). Tukey-Kramer post-hoc tests revealed that the “offline only” group differed from both other groups (“news feeds only” and “news feeds and online”) in age (both *p*-values < .001), in Conscientiousness (*p* < .001 versus the “news feeds only” group; *p* = .003 versus the “news feeds and online” group), and Neuroticism (*p* = .007 versus the “news feeds only” group; *p* = .046 versus the “news feeds and online” group). Descriptive statistics on differences in Conscientiousness and Neuroticism are presented in Figure 2. All analyses including the B-RWA-6 scale rely on groups of *n* = 49, *n* = 46, and *n* = 272 participants (“news feeds only”, “news feeds and online”, “offline only”).

**Table 3:** Multinomial logistic regression to predict group membership.

	News feeds and online (*n* = 46) (versus “news feeds only” (*n* = 49))		Offline only (*n* = 272) (versus “news feeds only” (*n* = 49))	
	Coefficient (SE)	p	Coefficient (SE)	p
Intercept	0.03 (.287)	.930	1.89 (.223)	<.001
Age	0.05 (.262)	.856	0.82 (.202)	<.001
Gender	-0.02 (.261)	.945	0.11 (.216)	.602
Extraversion	-0.16 (.231)	.493	-0.23 (.184)	.202
Agreeableness	-0.34 (.228)	.132	-0.11 (.185)	.551
Conscientiousness	0.22 (.234)	.347	0.51 (.189)	.007
Neuroticism	-0.17 (.244)	.495	-0.40 (.194)	.039
Openness	0.22 (.211)	.299	0.08 (.165)	.626
B-RWA-6	-0.27 (.235)	.246	0.10 (.188)	.592

*Note.* The “news feeds only” group was coded “1” (reference group), the “news feeds and online” group was coded “2” and the “offline only” group was coded “3”. The groups for these analyses contained *n* = 49, *n* = 46, and *n* = 272 participants (“news feeds only”, “news feeds and online”, “offline only”). The predictors (except gender; 0 = male, 1 = female) were z-standardized (in the complete sample) before including them in the model; hence, in *N* = 1,681 for age and the Big Five and in *n* = 1,397 for the B-RWA-6 (balanced short scale on authoritarian attitudes). The "news feeds only" group is not presented in the table as it is the reference group.

### 3.3 Associations between the number of news sources consumed and voting preferences

#### 3.3.1 Predicting the number of news sources consumed in total

The zero-inflation model predicting excessive zeros shows that the age (Estimate = -0.07, *SE* = .015, *z* = -4.69, *p* < .001), voting for the CDU/CSU (Estimate = -1.35, *SE* = .581, *z* = -2.33, *p* = .020), the SPD (Estimate = -1.50, *SE* = .719, *z* = -2.08, *p* = .038), the Grüne (Estimate = -1.39, *SE* = .433, *z* = -3.20, *p* = .001), and the Linke (Estimate = -1.49, *SE* = .708, *z* = -2.10, *p* = .036) are significant predictors. This indicates that younger age and not voting for the previously mentioned parties is associated with an increased chance that zeros occur because individuals do not consume news at all. The (zero-inflated) negative binomial (count) model is presented in Table 4 alongside some descriptive statistics. As can be seen in this table, age (positively), gender (negatively: implicating higher scores for males), and voting for the CDU/CSU, FDP, SPD, Grüne, and/or the Linke are significant predictors for the number of news sources consumed in total. The regression weights indicate that the predicted number of news sources consumed in total increases by 13% (exp(0.12) = 1.13) if age increases by one standard deviation (while holding all other variables constant), and decreases by 20% (exp(-0.22) = 0.80) for being female (while holding all other variables constant). Moreover, the predicted number of news sources consumed in total increases by 22% (exp(0.20) = 1.22) for voters of the CDU/CSU, increases by 27% (exp(0.24) = 1.27) for voters of the FDP, increases by 33% (exp(0.28) = 1.33) for voters of the SPD, increases by 36% (exp(0.30) = 1.36) for voters of the Grüne, and increases by 43% (exp(0.36) = 1.43) for voters of the Linke compared to the reference group of non-voters (all effects when holding all other variables in the model constant) (percentages are derived from the exact (unrounded) regression weights).

**Table 4:** (Zero-inflated) negative binomial model predicting the number of news sources consumed in total by age, gender, and current voting preferences.

	(Zero-inflated) negative binomial model				Descriptives	
	Estimate	SE	z	p	M	SD
Intercept	2.02	.073	27.79	<.001		
Age	0.12	.017	7.05	<.001		
Gender	-0.22	.034	-6.65	<.001		
AfD (*n* = 49)	0.06	.112	0.55	.582	7.24	5.10
Others (*n* = 129)	0.15	.087	1.67	.095	7.31	4.97
CDU/CSU (*n* = 192)	0.20	.081	2.44	.015	8.01	4.66
FDP (*n* = 85)	0.24	.094	2.58	.010	8.53	4.90
SPD (*n* = 117)	0.28	.087	3.22	.001	8.56	4.90
Grüne (*n* = 559)	0.30	.074	4.15	<.001	8.73	5.22
Linke (*n* = 118)	0.36	.087	4.11	<.001	9.39	5.42

*Note.* The groups with different current voting preferences are ordered in ascending order based on their mean number of news sources consumed in total. Age was z-standardized (in the complete Sample, hence, in *N* = 1,681) before included in the model; Gender was dummy coded as 0 = male, and 1 = female. Also, the variables on current voting preferences were dummy coded as 0 = would not vote respective party, and 1 = would vote respective party; “I would not vote” was used as reference group and, therefore, is not presented in the table (*n* = 91; *M* = 5.54, *SD* = 5.01). Results are derived from the sample of *n* = 1,340 participants. Log(theta) = 1.62, *p* < .001.

**Figure 3:**
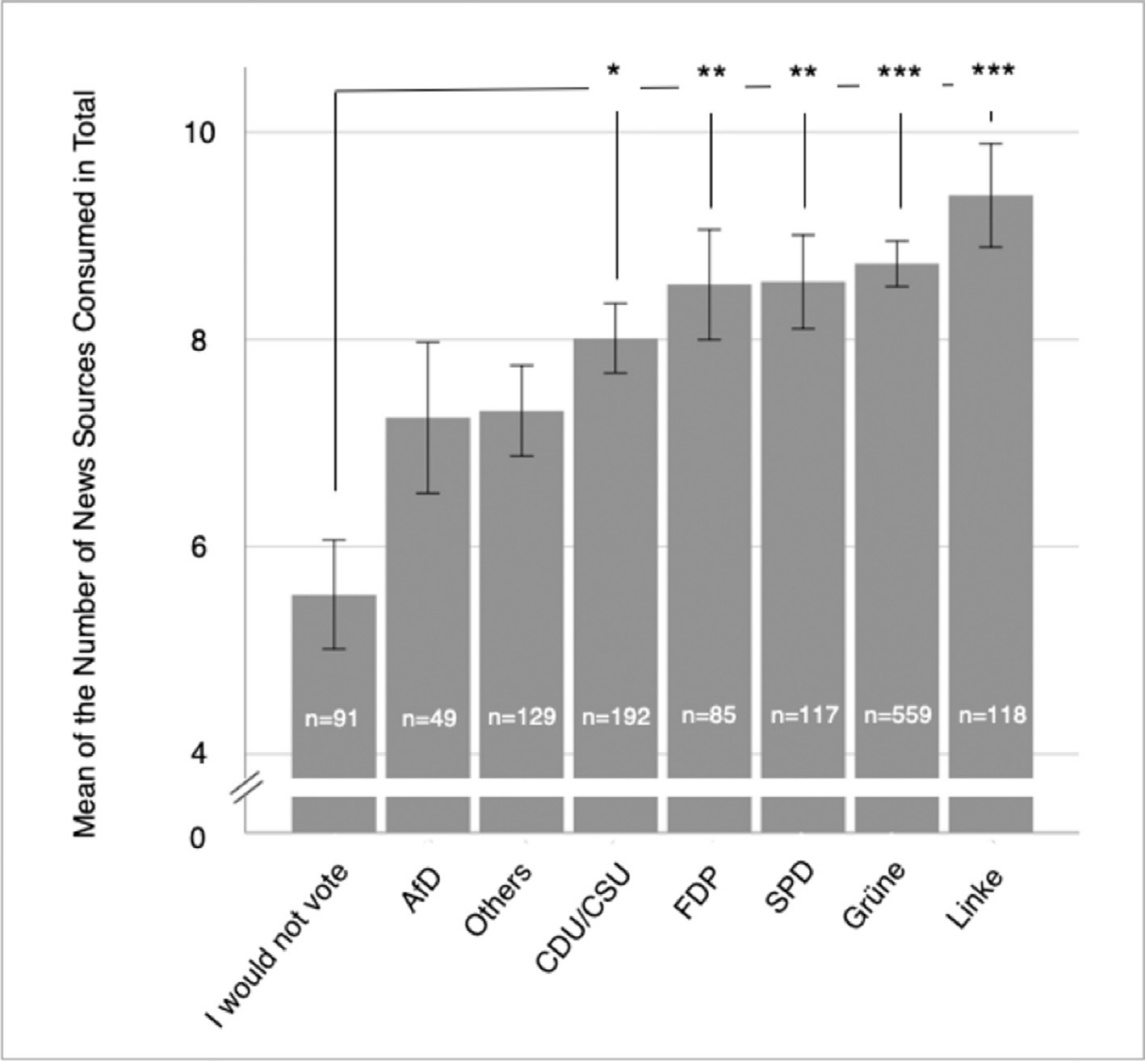
Mean Values and *SE*s (+/- 1 *SE*) of the number of news sources consumed in total split by voter groups; *p < .05, **p < .01, ***p < .001.

Descriptive statistics for the number of news sources consumed in total split by voter groups are also presented in Figure 3.

## 4 Discussion

The aim of the present work was to investigate whether (and to what extent) demographic variables, personality, and ideological attitude might influence if individuals put themselves at risk for ending up in a “filter bubble” and/or “echo chamber” online. Specifically, it was investigated whether the aforementioned variables influence the number of different news sources consumed. In turn, the number of news sources consumed is thought to be negatively associated with the risk for “filter bubbles” and “echo chambers”. Moreover, individuals who exclusively consume news via news feeds of social networking sites should be at highest risk to be caught in a “filter bubble” or “echo chamber”. Additional analyses revealed associations with voting preferences.

The results showed that in addition to age (positively) and gender (higher in males), Openness (positively) and especially RWA (negatively) predicted the number of news sources consumed in total (of note, in the Supplementary Material, also results on predictors of the summed scores of news sources consumed offline and online separately are presented). The present results regarding the positive association between Openness and the number of news sources consumed in total are partly in line with a previous study. In this previous study it was also found that Openness was positively associated with whether participants consumed news via TV and via the Internet (with regard to political information) within a seven-day period (Gerber et al., 2011). Such a positive link might be explained by the propensity of individuals scoring high in Openness to strive for new experiences and ideas and to discuss new ideas (Rammstedt and Danner, 2017). Overall, one might argue that high scores in Openness are associated with the proclivity for being stimulated by processing new information, including information on current news. This in turn might lead to a higher number of diverse news sources consumed. A significant effect was also found for the negative association between RWA and the number of news sources consumed in total. Another study reports that RWA was (among other variables) associated with selective interest in information fitting with one's own attitudes in a threat condition (Lavine et al., 2005). Such preferences to consume information (i. e. news) fitting with one's pre-existing attitudes could automatically diminish the number of news sources consumed; because only sources supporting one's own attitudes are used. This idea is supported by the present findings. Furthermore, the need for simple structure (a factor of need for closure), which also includes discomfort occasioned by ambiguity, has been positively associated with RWA in a previous study (van Hiel et al., 2004). This finding underlines the idea that individuals scoring high in RWA tend to avoid counter-attitudinal information and consequently, only consume a few news sources (potentially biased towards pre-existing attitudes).

Moreover, being a member of the group of participants consuming news exclusively via offline channels (TV, print, radio) was associated with the highest scores in Conscientiousness and (with a smaller effect size) lowest scores in Neuroticism compared to the groups of participants consuming news via news feeds only and via news feeds plus online news websites. As mentioned before, the Internet, and especially online social networking sites, pose a risk for the emergence of both “filter bubbles” and “echo chambers” (Bakshy et al., 2015; Nikolov et al., 2015; Zuiderveen Borgesius et al., 2016). On the contrary, at least the emergence of a “filter bubble” is not possible offline. Regarding Neuroticism, it seems like more depressed, anxious, and worrying individuals (Rammstedt and Danner, 2017) tend to consume news online and via news feeds only, therefore, putting themselves at higher risk for “filter bubbles” and “echo chambers”. An explanation for this association is speculative. However, as news consumed online and especially via social media might be highly personalized, only consuming news via these channels might be useful for more anxious individuals to avoid bad and threatening news. Additionally, it seems that especially individuals who describe themselves as hard working, careful, orderly, and proficient (Rammstedt and Danner, 2017) counteract the risk of ending up in a “filter bubble” and/or “echo chamber” by consuming news (exclusively) offline. However, it is also important to note that in the present sample of 1,681 participants, only 73 participants (4.34%) put themselves at high risk for being caught in a “filter bubble” and/or “echo chamber” online by exclusively consuming news via social networking sites, hence, highly personalized platforms. Most participants reported consuming news offline as well as online. These participants might be at some risk to end up in a “filter bubble” or “echo chamber” online. However, they also seem to counteract this risk by consuming news via various different online and offline channels (see for example results on media diversity and “echo chambers” (Dubois and Blank, 2018)). The small number of participants found to consume news only via news feeds of social networking sites also adds to the growing debate about the actual danger of “filter bubbles” and “echo chambers” on society (Flaxman et al., 2016; Zuiderveen Borgesius et al., 2016).

The current voting preference of participants was additionally investigated. It was found that, overall, many participants stated that they would vote for the “Grüne” party (Green Party (complete name actually: Coalition 90/Green Party); European party: European Green Party; focused on environmental and social issues). This finding mirrors opinion polls about this party in Germany before and shortly after the European elections in May 2019 (which is in the time of data collection for the present study) (Suhr, 2019). Participants who stated that they would not vote showed the lowest number of news sources consumed in total. Previous results also indicate that not being informed well is an important reason to not vote (ARD, 2020; Europäisches Parlament, 2020). Of note, no significant effect of voting preference for the “AfD” (Alternative for Germany; European party: European Alliance of Peoples and Nations; right-wing party) or “other” parties versus not voting on the number of news sources consumed in total could be observed. These results indicate that a person's news spectrum and therefore the potential to be in a “filter bubble” and/or “echo chamber” online is associated with voting preferences.

In addition to “filter bubbles” and “echo chambers”, another negative consequence of a narrow news spectrum has not been discussed so far. In detail, a low number of news sources consumed might also be associated with a lower ability to detect so called “fake news”. In a work by Lazer et al. (2018) “fake news” were defined as “fabricated information that mimics news media content in form but not in organizational process or intent. Fake-news outlets, in turn, lack the news media's editorial norms and processes for ensuring the accuracy and credibility of information […]” (Lazer et al., 2018, p. 1094). As such, it can be assumed that “fake news” might be easier to identify if various different news sources are consumed, because a comparison of the contents is possible; in turn, one does not need to rely on a few (potentially personalized/biased or even unreliable) news sources. Therefore, the association between the number of different news sources consumed and the ability to detect “fake news” represents an interesting new research topic.

Some limitations of the present study should be mentioned. First of all, participants were asked how many different news sources they consumed to assess the breadth of a person's news spectrum. This strategy was used because even if one news source would be highly personalized (hence, biased), the influence would not be very dramatic if several other not or less personalized (biased) news sources would be consumed. However, participants were not asked which kind of news sources they consumed or how often they consumed news at all. Moreover, the content of the news read, or whether more hard or soft/entertaining news are consumed, was not assessed. However, it is likely that the news content consumed differs between online (especially social networking sites) and offline sources (Harcup & O'Neill, 2017). Especially online there is also a high amount of unpredictability regarding which news items are read. This decision can be influenced by mechanisms such as clickbaiting or can be driven by overall public interests in a certain topic (e.g. when generally a lot of news on one topic is spread). Both factors are independent from one's personality and ideological attitude. Additionally, with the present research design it is not possible to separate the risk for “filter bubbles” versus “echo chambers” online. Overall, the actual existence and expense of “filter bubbles” and “echo chambers” could not be investigated. Instead, it can only be inferred based on the numbers of news sources consumed and based on previous literature (e.g. Dubois and Blank (2018)). Additionally, the present results are derived from a German-speaking sample potentially limiting the generalizability of findings to samples from other countries. This limited generalizability is especially true for the additional results on voting preferences. Moreover, by the categorization into “news feeds only”, “news feeds and online”, and “offline only” groups clearly a piece of information is lost and some groups were rather small. Therefore, a replication of the results is needed. Moreover, we again draw attention to the additional results on the prediction of the number of offline and online news sources consumed reported in the Supplementary Material. Lastly, personality is seen as a rather stable construct, whereas the number of different news sources consumed was only assessed for the last six months prior to participation. Accordingly, one might conclude that personality causally influences the number of news sources consumed. However, the present research design is of correlational nature. Therefore, it is not possible to prove causal directionality, which is a further limitation of the present study.

Despite these limitations, the present work relies on a general and heterogeneous population-based sample (see for example the large age range). Moreover, results are in line with previously published literature underlining the validity of the data and results.

In conclusion, the present study supports the idea that demographic variables (age, gender), personality (Openness), and ideological attitudes (RWA) might influence the number of different news sources consumed and the decision about whether to only consume news via news feeds of social networking sites (age (negatively), Conscientiousness (negatively), Neuroticism (positively)). Accordingly, these variables most likely also influence the risk of being caught in a “filter bubble” and/or “echo chamber” online. However, effect sizes were not large, leaving space for additional explanatory variables. Additionally, it should also be acknowledged that only 4.34% of participants in the present study reported only consuming news via news feeds of social networking sites. This finding suggests that overall the effects of potential “filter bubbles” and/or “echo chambers” online on society and democracy might not be as dramatic as expected several years ago. However, current voting preferences were also found to be associated with the number of news sources consumed: Individuals who would not vote reported the lowest number of news sources consumed closely followed by the group of voters of the right-wing party (“AfD” (Alternative for Germany; European party: European Alliance of Peoples and Nations); right-wing party) and “other” parties.

Finally, it seems inevitable to bring together computer science and digital footprint data with personality psychology for future research approaches (Montag and Elhai, 2019). This field of research is commonly known as psychoinformatics (Markowetz et al., 2014; Montag et al., 2016). By combining knowledge from both fields, it will be possible for future studies to causally investigate demographic variables, personality, ideological attitudes, and voting preferences alongside news consumption including the content of the news consumed as well as the objectively measured expense of “filter bubbles” and/or “echo chambers” on an individual level.

## Declarations

### Author contribution statement

Cornelia Sindermann, Christian Montag: Conceived and designed the experiments; Performed the experiments; Analyzed and interpreted the data; Wrote the paper.

Jon D. Elhai, Morten Moshagen: Analyzed and interpreted the data; Wrote the paper.

### Funding statement

This work was supported by a Heisenberg grant by the German Research Foundation under grant number DFG, MO2363/3-2.

### Competing interest statement

The authors declare no conflict of interest.

### Additional information

Data associated with this study has been deposited at https://osf.io/jxqvs/files/.

Supplementary content related to this article has been published online at https://doi.org/10.1016/j.heliyon.2020.e03214.
