# Supplementary material for: Age, gender, personality, ideological attitudes and individual differences in a person's news spectrum: how many and who might be prone to “filter bubbles” and “echo chambers” online?
Source: Heliyon. 2020 Jan 17;6(1):e03214. doi: 10.1016/j.heliyon.2020.e03214 (PMC7002846; doi:10.1016/j.heliyon.2020.e03214)
Supplement: Supplement [file mmc1.pdf]

Supplementary Material

Age, gender, personality, ideological attitudes and individual differences in a person's news spectrum: how many and who might be prone to "filter bubbles" and "echo chambers" online?

Cornelia Sindermann, Jon D. Elhai, Morten Moshagen, & Christian Montag

Data Cleaning Procedure

Participants who made implausible specifications about their age (e.g. age of 6 years or lower or age of above 800 years; n=22) were excluded. Also, participants who reported to have consumed more than 1000 different news sources via one channel (e.g. via TV) within the past 6 months prior to participation were excluded (n=3). The same was true for participants who reported the same response option consecutively throughout at least one of the questionnaire measures (see section "Materials" in the Main Manuscript) (n=32). After exclusion of these participants, who seemed to evidence careless or insufficient motivation in responding, further outliers with regard to the numbers of news sources consumed in total, offline, and online (see description in the Main Manuscript) were identified by means of the formula by Tukey (1977) (scoring lower than [25th-Quantil - (1.5×(75th-Quantil - 25th-Quantil))] or higher than [75th-Quantil + (1.5×(75th-Quantil - 25th-Quantil))]) and excluded (n=146). Outliers in these variables were excluded as the distributions were extremely skewed. Additionally, there were several participants who still reported extreme and implausible scores on these variables before exclusion of outliers according to the formula by Tukey (1977) (e.g. to have consumed more than 368 different news sources within the past six months prior to participation). Lastly, also participants younger than 12 years were excluded given that participation was explicitly only allowed from age 12, provided that the parents or the legal guardian agreed on participation (n=10).

German Parties, which were under Investigation

Supplementary Table 1. Short description of some characteristics of German parties, which were under investigation in the present study.

Party	Information
**CDU / CSU**	Two parties with conservative, liberal, and Christian-social attitudes (https://www.bpb.de/politik/grundfragen/parteien-in-deutschland/cdu/42058/kurz-und-buendig; https://www.bpb.de/politik/grundfragen/parteien-in-deutschland/csu/)
**SPD**	Core values are: Freedom, Fairness, Solidarity (https://www.bpb.de/politik/grundfragen/parteien-in-deutschland/spd/)
**Grüne**	Emerged from the protest against environmental destruction, the use of nuclear energy, and nuclear armament (https://www.bpb.de/politik/grundfragen/parteien-in-deutschland/gruene/)
**FDP**	Economic liberal positions and a restrictive stance in refugee and European policies (https://www.bpb.de/politik/grundfragen/parteien-in-deutschland/fdp/)
**Linke**	Roots both in the labor union-related environment and the protest against the social policy of the 2000s (https://www.bpb.de/politik/grundfragen/parteien-in-deutschland/die-linke/)
**AfD**	Restrictive positions on immigration policy, a conservative social policy, and an anti-establishment orientation (https://www.bpb.de/politik/grundfragen/parteien-in-deutschland/afd/)

Zero-Order Bivariate Correlations between the Big Five and Right-Wing Authoritarianism

As can be seen in Supplementary Table 2, especially Openness of the Big Five inventory (BFI) is moderately negatively related to the B-RWA-6 scale (measuring Right-Wing Authoritarianism). Agreeableness is weakly negatively and Conscientiousness weakly positively related to the B-RWA-6 scale.

Supplementary Table 2. Zero-order Pearson correlations between the BFI and the B-RWA-6 scales.

	B-RWA-6 total
Extraversion	r=-.01, p=.623
Agreeableness	r=-.15, p<.001
Conscientiousness	r=.12, p<.001
Neuroticism	r=.04, p=.135
Openness	r=-.24, p<.001

Note. Correlations are derived from the sample size of n=1,397 participants.

Age and Gender and the Number of News Magazines Consumed Offline and Online

Age correlated significantly with the number of news magazines consumed offline and online (offline: $r=.33$, $p<.001$; online: $r=.05$, $p=.048$). Significant gender differences were also found in the summed scores of news magazines consumed offline and online. Males (offline: $M=5.77$, $SD=4.39$; online: $M=3.20$, $SD=2.78$) scored significantly higher in both scores compared to females (offline: $M=4.86$, $SD=3.88$; online: $M=2.48$, $SD=2.51$) (offline: $t(994.93)=4.14$, $p<.001$, Hedge's $g = .22$; online: $t(1010.96)=5.14$, $p<.001$, Hedge's $g = .28$).

Partial Correlations between the BFI and the B-RWA-6 Scales and the Number of News Sources Consumed in Total, Offline, and Online

The partial Pearson correlations (corrected for age) as presented in Supplementary Table 3 show that the number of news sources consumed in total is positively related to Extraversion and especially Openness. On the other hand, the number of news sources consumed in total is negatively related to the B-RWA-6 scale. Moreover, the number of news sources consumed offline is significantly positively related to Extraversion and Conscientiousness, and significantly negatively related to Neuroticism. On the other hand, the number of news sources consumed online is significantly positively related to Openness and significantly negatively related to Conscientiousness and especially the B-RWA-6 scale. However, effect sizes are rather small. Correlations separately for males and females are not presented because no significant differences were found for the partial Pearson correlations.

Supplementary Table 3. Partial Pearson correlations of the numbers of news sources consumed with the BFI and the B-RWA-6 scales.

	Total number	Offline number	Online number
Extraversion	r_p=.06, p=.019	r_p=.08, p<.001	r_p=-.01, p=.598
Agreeableness	r_p=.02, p=.382	r_p=.03, p=.289	r_p=.00, p=.904
Conscientiousness	r_p=.02, p=.472	r_p=.06, p=.022	r_p=-.05, p=.047
Neuroticism	r_p=-.04, p=.103	r_p=-.05, p=.043	r_p=(-).00, p=.878
Openness	r_p=.10, p<.001	r_p=.04, p=.076	r_p=.12, p<.001
B-RWA-6[1]	r_p=-.11, p<.001	r_p=-.02, p=.553	**r_p=-.18, p<.001**

Note. All correlations are corrected for age. The columns about total number, offline number, and online number refer to the summed scores of numbers of news sources consumed in total, offline, and online, respectively. [1] Values of the B-RWA-6 (balanced short scale on authoritarian attitudes) correlations are derived from the sample size n=1,379 participants (instead of N(total sample)=1,681).

Predicting the Number of News Sources Consumed Offline

The zero-inflation model predicting excessive zeros shows that in addition to the intercept, also age (Estimate=-0.77, SE=.104, z=-7.35, p<.001) and Conscientiousness (Estimate=-0.29, SE=.088, z=-3.35, p<.001) significantly predict excessive zeros. This indicates that younger age and lower Conscientiousness are associated with an increased chance that the "0" in the data is due to the fact that people do not consume offline news sources at all. The (zero-inflated) negative binomial (count) model is presented in Supplementary Table 4. As can be seen in Supplementary Table 4, age (positively) and gender (negatively: implying higher scores for males) are the significant predictors for the number of news sources consumed offline. The regression weights indicate, that the predicted number of news sources consumed offline increases by 15% (exp(0.14)=1.15) if age is increased by one standard deviation (while holding all other variables constant), and decreases by 16% (exp(-0.17)=0.84) for being female (while holding all other variables constant).

Supplementary Table 4. (Zero-inflated) negative binomial model predicting the number of news sources consumed offline.

	Estimate	SE	z	p
Intercept	1.92	.030	64.80	<.001
Age	0.14	.017	7.98	<.001
Gender	-0.17	.037	-4.69	<.001
Extraversion	0.04	.019	1.91	.056
Agreeableness	-0.02	.019	-0.81	.417
Conscientiousness	-0.01	.019	-0.52	.600
Neuroticism	0.01	.020	0.36	.720
Openness	0.02	.018	1.22	.224
B-RWA-6	(-)0.00	.017	-0.06	.950

Note. n=1,397. Gender was dummy coded as 0=male, and 1=female. The predictors (except gender) were z-standardized (in the complete sample) before including them in the model; hence in N=1,681 for age and the Big Five and on n=1,397 for the B-RWA-6 (balanced short scale on authoritarian attitudes). Log(theta)=1.88, p<.001.

Predicting the Number of News Sources Consumed Online

The zero-inflation model predicting excessive zeros shows that next to the intercept also gender (Estimate=0.57, SE=.216, z=2.64, p=.008), Extraversion (Estimate=-0.23, SE=.099, z=-2.27, p=.023), and Neuroticism (Estimate=-0.23, SE=.110, z=-2.07, p=.038) significantly predict excessive zeros. This indicates that being female, lower Extraversion, and lower Neuroticism are associated with an increased chance that the “0” in the data is due to the fact that people do not read news online at all. As can be seen in Supplementary Table 5, gender (negatively: implicating higher scores for males), Extraversion (negatively), Openness (positively), and the B-RWA-6 scale (negatively) are the significant predictors of the number of news sources consumed online. For a better interpretation, the exact estimates indicate that the predicted number of news sources consumed online decreases by 12% (exp(-0.13)=0.88) for being female (while holding all other variables constant), decreases by 7% (exp(-0.07)=0.93) for one standard deviation increase in Extraversion (while holding all other variables constant), increases by 6% (exp(0.06)=1.06) for one standard deviation increase in Openness (while holding all other variables constant), and decreases by 10% (exp(-0.11)=0.90) for one standard deviation increase in the B-RWA-6 scale (while holding all other variables constant).

Supplementary Table 5. (Zero-inflated) negative binomial model predicting the number of news sources consumed online.

	Estimate	SE	z	p
Intercept	1.31	.041	32.21	<.001
Age	-0.01	.026	-0.45	.650
Gender	-0.13	.052	-2.58	.010
Extraversion	-0.07	.026	-2.69	.007
Agreeableness	-0.03	.025	-1.19	.235
Conscientiousness	-0.04	.026	-1.52	.128
Neuroticism	-0.04	.028	-1.32	.187
Openness	0.06	.026	2.39	.017
B-RWA-6	-0.11	.024	-4.58	<.001

Note. n=1,397. Gender was dummy coded as 0=male, and 1=female. The predictors (except gender) were z-standardized (in the complete sample) before including them in the model; hence in N=1,681 for age and the Big Five and on n=1,397 for the B-RWA-6 (balanced short scale on authoritarian attitudes). Log(theta)=1.57, p<.001.

Discussion on the Different Predictors of Numbers of News Sources Consumed Offline and Online

Whereas neither any of the personality variables nor RWA significantly predicted the number of news sources consumed offline, the number of news sources consumed online was significantly predicted by Extraversion (negatively), Openness (positively), and RWA (negatively) (next to gender). The result regarding Extraversion and its negative association with online news consumption is supported by a study in which, among others, also a negative (but non-significant) association of Extraversion is reported with an item about whether participants consumed Internet news (for political information) within a seven-day period (Gerber, Huber, Doherty, & Dowling, 2011). However, in this previous study Extraversion was significantly positively related to reading newspapers, whereas in the present study, a positive association with the number of news sources consumed offline was only found in the correlational analyses, however, not in the (zero-inflated) negative binomial models. Also the present results regarding Openness are partly in line with the previous study, in which it was also found that Openness was positively associated with whether participants consumed news via TV and on the Internet (with regard to political information; no versus yes) within a seven-day period (Gerber et al., 2011). However, the positive association between Openness and the number of news sources consumed offline was only found descriptively (but was not significant) in the present study. Overall, it needs to be mentioned that the number of news sources consumed online mostly relies on the number of news websites visited. This is due to the fact that reading news on news feeds of social networking sites was only assessed via a “no versus yes” question (but the number of different news feeds consumed was not assessed). Moreover, it was not asked, for example, whether news aggregators were used, which should be higher personalized as compared to news websites.

Personality Scores and Ideological Attitude in the Different Voter Groups

As can be seen in Supplementary Table 6, the group of participants stating that they would not vote showed descriptively the lowest scores in all BFI scales except in Conscientiousness and Neuroticism compared to the other groups. Participants who stated that they would vote the “Grüne” party reported descriptively the highest scores in Agreeableness compared to the other groups. Lastly, participants who stated that they would vote the CDU/CSU showed descriptively the highest scores in Conscientiousness and the lowest scores in Neuroticism compared to the other groups. The group of participants who would vote for the AfD showed significantly higher scores in the B-RWA-6 scale compared to all other groups. A multivariate ANOVA revealed a significant effect of the voter group factor (multivariate effect: $F(42,7992)=9.07$, $p<.001$). The ANOVAs showed significant differences between the voter groups in each BFI scale. Descriptive statistics and results of Tukey-Kramer post-hoc tests are presented in Supplementary Table 6.

Supplementary Table 6. Descriptive statistics of the BFI and the B-RWA-6 scales split by groups of voters of different parties.

	Extraversion	Agreeableness	Conscientiousness	Neuroticism	Openness	RWA
AfD (n=49)	3.35 (0.75)	3.39 (0.55)	3.67 (0.58)[f]	2.92 (0.72)	3.50 (0.67)	3.49 (0.59) [a,b,c,d,e,f,g]
CDU/CSU (n=192)	3.42 (0.81)	3.50 (0.54)	3.78 (0.66)[a,b,c]	2.77 (0.79)[a,b,c]	3.40 (0.58)[a,b,c]	3.09 (0.54) [a,h,i,j,k,l]
FDP (n=85)	3.47 (0.70)	3.50 (0.54)	3.56 (0.63)	2.92 (0.81)	3.64 (0.55)[b,e]	2.84 (0.56) [c,j,m,n,o]
Grüne (n=559)	3.44 (0.78)[a]	3.63 (0.54)[a]	3.65 (0.64)[d,e]	2.97 (0.79)	3.68 (0.56)[a,d]	2.51 (0.61) [g,i,m,p,q,r]
I would not vote (n=91)	3.13 (0.87)[a]	3.32 (0.61)[a,b]	3.41 (0.82)[c,e]	3.09 (0.87)[c]	3.36 (0.64)[d,e,f]	3.15 (0.56) [f,o,r,s,t,u]
Linke (n=118)	3.30 (0.75)	3.58 (0.57)[b]	3.30 (0.68)[a,d,f]	3.07 (0.82)[a]	3.66 (0.55)[c,f]	2.35 (0.67) [d,k,n,t,v,w]
Others (n=129)	3.27 (0.78)	3.53 (0.55)	3.49 (0.61)[b]	3.08 (0.84)[b]	3.55 (0.60)	2.76 (0.62) [e,l,q,u,w]
SPD (n=117)	3.46 (0.79)	3.53 (0.54)	3.56 (0.69)	3.04 (0.78)	3.52 (0.63)	2.85 (0.62) [b,h,p,s,v]

Note. B-RWA-6: balanced short scale on authoritarian attitudes The groups with different current voting preferences are ordered in alphabetical order. Results are derived from the sample of n=1,340 participants. The same letters indicate that the two groups significantly differed in their mean according to the post-hoc tests. Results of the ANOVAs: Extraversion: $F(7,1332)=2.69$, $p=.009$, η^2(partial)=.014; Agreeableness: $F(7,1332)=4.81$, $p<.001$, η^2(partial)=.025; Conscientiousness: $F(7,1332)=7.56$, $p<.001$, η^2(partial)=.038; Neuroticism: $F(7,1332)=2.79$, $p=.007$, η^2(partial)=.014; Openness: $F(7,1332)=7.44$, $p<.001$, η^2(partial)=.038, RWA: $F(7,1332)=46.69$, $p<.001$, η^2(partial)=.197.

References

Gerber, A. S., Huber, G. A., Doherty, D., & Dowling, C. M. (2011). Personality traits and the consumption of political information. *American Politics Research*, *39*(1), 32–84. https://doi.org/10.1177/1532673X10381466

Tukey, J. W. (1977). *Exploratory data analysis* (1st ed.). Reading, Mass: Pearson.
